# Functional Divergence in the Role of N-Linked Glycosylation in Smoothened Signaling

**DOI:** 10.1371/journal.pgen.1005473

**Published:** 2015-08-20

**Authors:** Suresh Marada, Gemma Navarro, Ashley Truong, Daniel P. Stewart, Angela M. Arensdorf, Sigrid Nachtergaele, Edgar Angelats, Joseph T. Opferman, Rajat Rohatgi, Peter J. McCormick, Stacey K. Ogden

**Affiliations:** 1 Department of Cell & Molecular Biology, St. Jude Children’s Research Hospital, Memphis, Tennessee, United States of America; 2 Department of Biochemistry and Molecular Biology, Centro de Investigacion Biomedica en Red sobre Enfermedades Neurodegenerativas (CIBERNED)University of Barcelona, Barcelona, Spain; 3 Summer Plus Program, Rhodes College, Memphis, Tennessee, United States of America; 4 Departments of Medicine and Biochemistry, Stanford University School of Medicine, Stanford, California, United States of America; 5 School of Pharmacy, University of East Anglia, Norwich, Norfolk, United Kingdom; University of Miami, UNITED STATES

## Abstract

The G protein-coupled receptor (GPCR) Smoothened (Smo) is the requisite signal transducer of the evolutionarily conserved Hedgehog (Hh) pathway. Although aspects of Smo signaling are conserved from *Drosophila* to vertebrates, significant differences have evolved. These include changes in its active sub-cellular localization, and the ability of vertebrate Smo to induce distinct G protein-dependent and independent signals in response to ligand. Whereas the canonical Smo signal to Gli transcriptional effectors occurs in a G protein-independent manner, its non-canonical signal employs Gαi. Whether vertebrate Smo can selectively bias its signal between these routes is not yet known. N-linked glycosylation is a post-translational modification that can influence GPCR trafficking, ligand responsiveness and signal output. Smo proteins in *Drosophila* and vertebrate systems harbor N-linked glycans, but their role in Smo signaling has not been established. Herein, we present a comprehensive analysis of *Drosophila* and murine Smo glycosylation that supports a functional divergence in the contribution of N-linked glycans to signaling. Of the seven predicted glycan acceptor sites in *Drosophila* Smo, one is essential. Loss of N-glycosylation at this site disrupted Smo trafficking and attenuated its signaling capability. In stark contrast, we found that all four predicted N-glycosylation sites on murine Smo were dispensable for proper trafficking, agonist binding and canonical signal induction. However, the under-glycosylated protein was compromised in its ability to induce a non-canonical signal through Gαi, providing for the first time evidence that Smo can bias its signal and that a post-translational modification can impact this process. As such, we postulate a profound shift in N-glycan function from affecting Smo ER exit in flies to influencing its signal output in mice.

## Author Summary

N-linked glycosylation is a post-translational modification occurring on membrane proteins such as G protein-coupled receptors (GPCR). Smoothened (Smo) is a GPCR that functions as the signal transducer of the Hedgehog (Hh) pathway. We used a mutagenesis approach to assess the role of N-glycans in Smo signaling in two genetic models for Hh pathway activity, *Drosophila* and mouse. In doing so, we discovered a divergence in glycan function between them. We mapped an essential N-glycan acceptor site that when lost in *Drosophila*, triggered ER retention, altered Smo protein stability and blunted its signaling capacity. Conversely, ER exit of the murine protein was unaffected by glycan loss, as was its ability to traffic and induce a G protein-independent signal to activate Hh target genes. However, the ability of vertebrate Smo to induce a distinct G protein-dependent signal was lost. This suggests that N-linked glycosylation may influence signal bias of vertebrate Smo to favor one signal output over the other. We therefore propose that the role of this conserved post-translational modification may have been repurposed from governing Smo ER exit in the fly to influencing effector route selection in vertebrates.

## Introduction

The Hedgehog (Hh) signaling pathway contributes to developmental patterning and adult tissue homeostasis, and when corrupted, can contribute to tumor initiation and maintenance [1–3]. Originally identified in *Drosophila*, the Smoothened (Smo) protein is an evolutionarily conserved G protein-coupled receptor (GPCR) that functions as the signal transducer of the Hh cascade [4–6]. Its activity is indirectly regulated by the Hh ligand through the receptor Patched (Ptc), which in the absence of Hh, blocks Smo signaling by inhibiting its accumulation on the plasma membrane in *Drosophila* or in the primary cilium in vertebrates [7,8]. As such, in the absence of Hh, Smo is localized predominantly to recycling endosomes where it does not signal [9–11]. The mechanism by which Ptc controls Smo localization and signaling has not yet been defined, however a predominant model postulates that Ptc governs the availability of an unknown Smo ligand [12,13]. In vertebrate systems this ligand is likely to be a sterol-like compound because 20(S)-hydroxy cholesterol (20(S)-OHC) and the steroidal alkaloid cyclopamine are both modulators of Smo activity [14,15]. Naturally occurring compounds modulating insect Smo activity have yet to be discovered. However, it is well established that Hh controls pathway activation in both vertebrates and invertebrates by binding to Ptc and its associated co-receptors [12,16–19]. This binding induces a Ptc conformation shift that triggers its internalization and lysosomal degradation, thereby allowing Smo to translocate to its active signaling location [9,20]. From there, Smo communicates with its downstream effectors to induce intracellular signaling. In flies and canonical vertebrate signaling, this culminates in activation Hh target gene expression through the Gli/Ci family of transcription factors [11,21,22].

Although both *Drosophila* and vertebrate Smo proteins are capable of activating Gαi heterotrimeric G proteins in response to Hh, the role of Gαi in Smo signaling has evolved [6,23]. In vertebrates, Smo-mediated Gαi activation appears to be dedicated to induction of a distinct, non-canonical Hh signal that can alter intracellular Ca^2+^ levels to modulate phospholipase C activity or induce RhoA and Rac to govern cell migration [23–25]. This suggests that, in some contexts, vertebrate Smo might display biased signaling whereby one effector route is favored over the other [26,27]. Although it is not yet possible to predict how signal bias is controlled for a given GPCR, it is possible that post-translational receptor modifications affecting ligand sensitivity and responsiveness may contribute [28,29].

A common post-translational modification occurring on GPCRs is N-linked glycosylation, which can affect a multitude of processes including receptor folding, trafficking, stability, cell-surface localization, ligand binding and ligand responsiveness [30–34]. Although N-linked glycosylation occurs in both insect and vertebrate systems, clear differences exist in oligosaccharide processing and glycan complexity [35]. In this report, we interrogate N-linked glycosylation of Smo proteins in fly and murine systems to determine whether differential glycosylation patterns between the two have distinct effects on signaling activity. We describe a clear evolutionary divergence in the role of N-glycans for Smo activity and postulate that with the emergence of non-canonical Smo signaling in vertebrates, the role of glycosylation in its activity evolved from assisting in protein folding and ER exit in flies to a novel role of influencing signal output in vertebrates.

## Results

### Identification of N-linked glycosylation sites in Smo proteins

**Fig 1:**
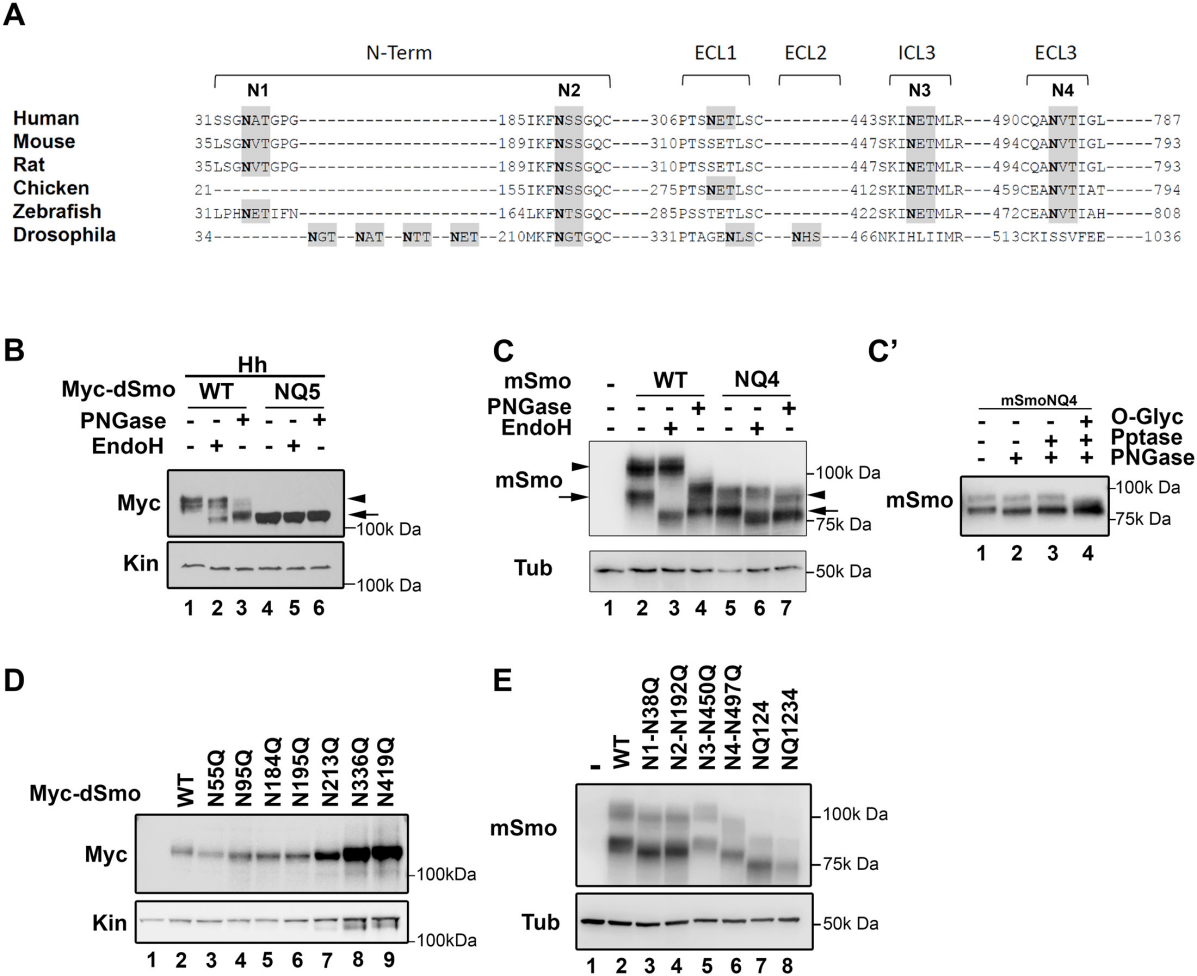
Identification of Smo N-linked glycosylation sites. **A**. A multiple sequence alignment of Smo proteins from different phyla are shown. Consensus sequences for N-linked glycosylation are highlighted in gray and the Asn acceptor residues are bold. Sites conserved across vertebrate proteins are indicated as N1-N4. The predicted *D*. *melanogaster* sites are not tightly conserved with vertebrates. **B**. Drosophila Smo is N-glycosylated. Cell lysates prepared from Cl8 cells expressing Hh with wild type or NQ5 dSmo proteins were treated with the indicated deglycosylating enzymes. Wild type Smo demonstrated ER (arrow) and post-ER (arrowhead) glycosylation species. NQ5 migrated similarly to the fully deglycosylated species under all conditions (arrow). **C**. Mouse Smo is N-glycosylated. Cellular lysates from *Smo-/-* cells stably expressing mSmoWT or mSmoNQ4 were treated with deglycosylating enzymes and subjected to SDS-PAGE and western blot. mSmoWT resolves as two distinct forms (lane 2). The arrow marks the ER form and the arrowhead indicates the post-ER form. mSmoNQ4 migrates in SDS-PAGE similarly to the PNGase-treated wild type protein (lanes 4–5). **C’**. mSmoNQ4 is O-glycosylated. Lysates were prepared from NIH3T3 cells expressing mSmoNQ4 and subjected to lambda phosphatase, PNGase and O-glycosidase/neuraminidase treatments. The upper band collapsed upon O-glycosidase/neuraminidase treatment. **D**. Expression of individual N to Q dSmo mutants. The indicated N to Q dSmo mutants were expressed in Cl8 cells and cell lysates were analyzed by SDS-PAGE and western blot against the Myc tag. Kin serves as loading control. **E**. Extracellular mSmo consensus sites are N-glycosylated. Mutation of individual extracellular mSmo glycosylation sites induced faster mobility on SDS-PAGE. mSmoN450Q migrated similarly to SmoWT. For western blots, mSmo was detected using anti-Smo and dSmo with anti-Myc. Kinesin (Kin) and Tubulin (Tub) were blotted for loading controls.

To identify the predicted N-linked glycosylation sites in Smo that are conserved across phyla, Smo protein sequences from human, mouse, rat, chicken, zebrafish and fly were analyzed using NetNGlyc prediction software (Fig 1A). This identified four high confidence motifs (NxS/T), one intracellular and three extracellular, that are conserved across the vertebrate Smo proteins (Fig 1A, N1-N4). These are localized to the extracellular amino-terminal region flanking the cysteine rich domain (CRD), in extracellular loop EC3 and in intracellular loop IC3 (Fig 1A and S1B Fig). The predicted N-glycosylation pattern differs significantly for *Drosophila* Smo (dSmo), which contains seven predicted consensus sites: five within its amino-terminal CRD, one in EC1 and one in EC2 (Fig 1A and S1A Fig). Multiple sequence alignment of all sequenced *Drosophila* Smo proteins using Clustal W software revealed that of these seven predicted sites, five are conserved across *Drosophila* species: N95, N184, N195, N213 and N336 (S1C Fig).

To determine whether the identified motifs harbored N-glycosylation moieties, compound Smo mutants harboring N to Q alterations of the conserved acceptor sites in dSmo (N95Q, N184Q, N195Q, N213Q and N336Q, hereafter referred to as dSmoNQ5) and mouse Smo (N38Q, N192Q, N450Q and N497Q, mSmoNQ4) were generated and tested for EndoH and PNGase sensitivity (Fig 1B and 1C). PNGase cleaves all N-linked glycans including mannose rich, hybrid and complex oligosaccharide species. EndoH recognizes and cleaves N-linked mannose rich oligosaccharides that are present on ER-resident proteins, but does not cleave the highly processed complex oligosaccharides that are added in post-ER compartments. Cell lysates prepared from Drosophila Clone 8 (Cl8) cells expressing Hh with wild type or NQ5 dSmo proteins were incubated with the indicated deglycosylating enzymes. Wild type dSmo was sensitive to both of these deglycosylating enzymes, revealing an EndoH-resistant post-ER pool (Fig 1B, arrowhead) and an EndoH-sensitive ER pool (arrow). The residual shift evident following PNGase treatment is likely due to Hh-induced phosphorylation (lane 3 and [36]). dSmoNQ5 migrated similarly to the fully deglycosylated, PNGase treated species under all conditions (Fig 1B lanes 4–6 compared to 3), supporting that the mutant is N-glycan deficient. Consistent with the established role of N-linked glycosylation in protein turnover [37], dSmoNQ5 protein levels appeared higher than wild type (Fig 1B, compare lanes 1 and 4). Half-life analysis confirmed that NQ5 was indeed stabilized, demonstrating a half-life of ~4 hours compared to ~1 hour for wild type (S1D Fig).

For mSmo studies, *Smo-/-* cells were stably transfected with vector control, wild type or NQ4 mSmo expression plasmids in order to minimize transfection and expression variability across experiments. To minimize over-expression effects, stable lines showing the lowest equivalent levels of wild type and NQ4 mSmo proteins were selected (Fig 1C). Deglycosylating assays were performed on lysates prepared from *Smo-/-* cells stably transfected with plasmids encoding wild type or NQ4 mutant mSmo (Fig 1C). mSmoNQ4 demonstrated a faster migration pattern than wild type, consistent with it having decreased glycosylation (Fig 1C compare lanes 2 and 5). Whereas wild type mSmo possessed both simple N-linked glycans (left arrow) and complex post-ER N-linked glycans (left arrowhead), the NQ4 mutant did not (Fig 1C compare lanes 2–4 with 5–7). NQ4 demonstrated a banding pattern similar to PNGase-treated wild type Smo under its basal conditions, and did not collapse upon EndoH or PNGase treatments (lane 4 compared to 5–7). In addition to possessing N-linked glycosylation, mSmo also harbors O-linked glycan modifications [36]. Accordingly, the upper band evident for mSmoNQ4 (Fig 1C, lane 7 right arrowhead) was unaffected by lambda phosphatase treatment, but collapsed upon O-glycosidase/neuraminidase treatment (Fig 1C’). Thus, stripping mSmoNQ4 of its N-glycans does not impact O-linked glycan addition.

To narrow down which of the predicted N-glycan acceptor sites in the two proteins harbor modifications, single N to Q mutations were generated for dSmo and mSmo proteins, and their migration assessed by SDS-PAGE (Fig 1D and 1E). Each of the dSmo mutants exhibited a modest migration shift, consistent with each of the predicted acceptor sites harboring glycan modifications (Fig 1D, lane 2 compared to 3–6). For mSmo, 3 of the 4 predicted sites displayed altered migration (Fig 1E). Consistent with N-glycosylation machinery in the ER lumen not having access to the cytoplasmic domains of mSmo, the predicted site in IC3 (N3) did not shift upon N to Q mutation (compare lanes 2 and 5). Conversely, each of the three remaining sites demonstrated size collapse and faster gel migration upon N to Q mutation (Fig 1E compare lane 2 with 3, 4 and 6).

### Glycan modifications are essential for *Drosophila* Smo function

**Fig 2:**
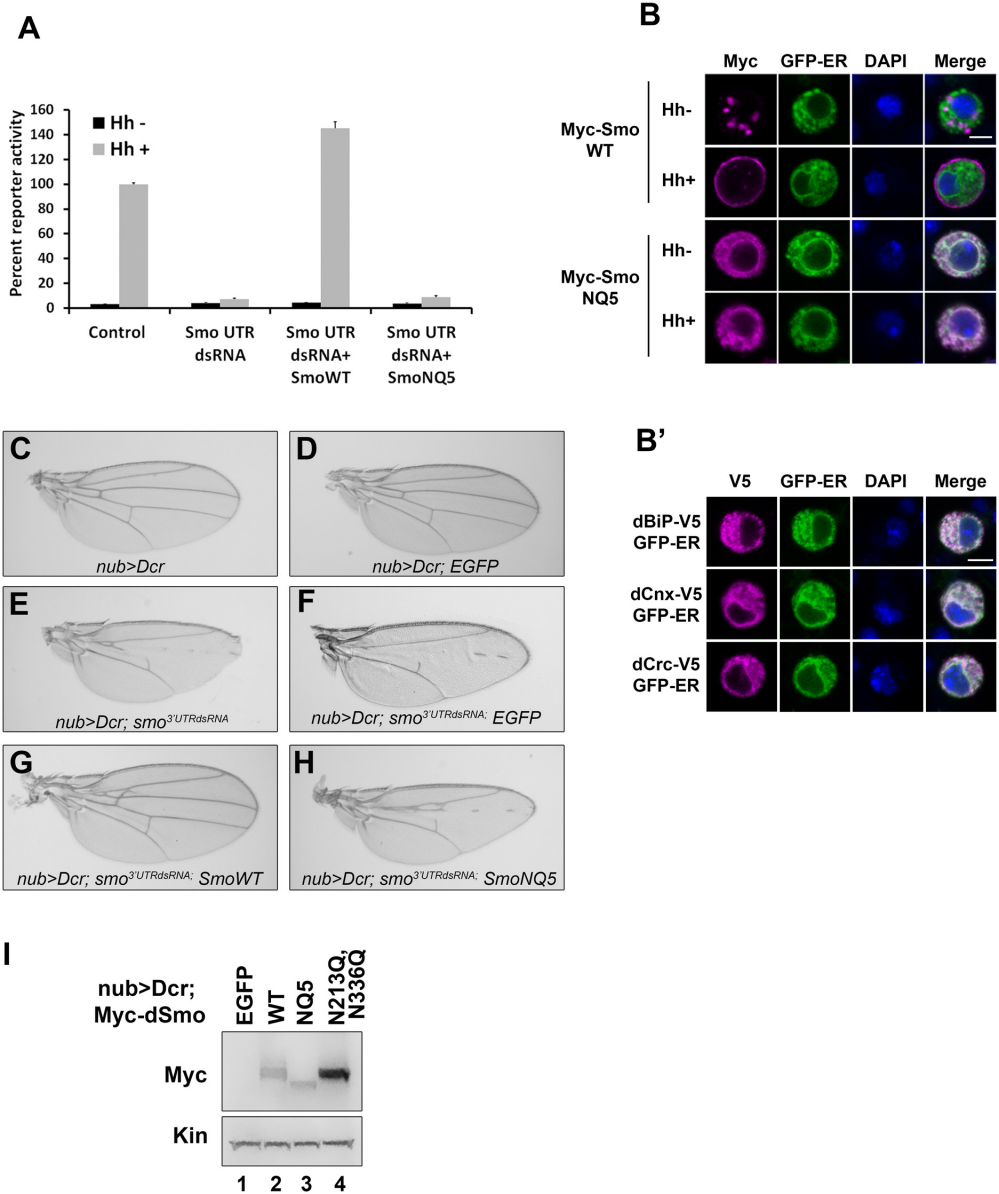
N-linked glycans are required for dSmo trafficking and activity. **A.** dSmoNQ5 does not signal *in vitro*. Cl8 cells were transfected with control or *smo 5’UTR* dsRNA, the *ptcΔ136-luciferase* reporter, *pAc-renilla* control, *pAc-myc-smoWT* or *NQ5*, and *pAc-hh* or empty vector control. Hh-induced reporter activity (gray bars) was ablated by knockdown of endogenous *smo* and rescued by dSmo cDNA lacking UTR sequence for wild type, but not for dSmoNQ5. **B-B’**. dSmoNQ5 demonstrates altered sub-cellular localization. Cl8 cells expressing Calreticulin-EGFP-KDEL ER marker (GFP-ER, green) and Myc-SmoWT or NQ5 in the presence or absence of Hh were imaged by immunofluorescence microscopy. Wild type dSmo (anti-Myc, magenta) localized to puncta that did not overlap with the ER marker in the absence of Hh, and translocated to the plasma membrane in response to Hh. The NQ5 mutant overlapped with the ER marker under both conditions. DAPI (blue) marks the nucleus. Scale bar is 5 μm (upper right box). **B’**. GFP-ER colocalizes with V5 tagged BiP, Calnexin (Cnx) and Calreticulin (Crc). DAPI marks the nucleus. **C-H.** dSmoNQ5 does not signal *in vivo*. Transgenes encoding wild type (G) or NQ5 (H) dSmo proteins were expressed in the *nubbin>dicer;smo* ^*3’UTR*^ background (E). Whereas wild type Smo could rescue the loss of function phenotype induced by *smo* ^*3’UTR*^, dSmoNQ5 could not (G-H compared to F and C-D, control). *UAS-EGFP* was expressed in the *nubbin>dicer;smo* ^*3’UTR*^ background and serves as a control for normalized transgene dosage (F). **I.** Wild type and NQ5 dSmo proteins are present at similar protein levels in wing imaginal disc tissue lysate. The dSmoN213Q,N336Q protein level is higher.

Having established that both fly and mouse Smo proteins harbor N-linked glycans, we next wanted to determine the contribution of glycans to Smo function. To interrogate the fly protein, the ability of dSmoNQ5 to rescue Smo-dependent activation of a Hh reporter construct (*ptcΔ136-luciferase*) in Cl8 cells following knockdown of endogenous *smo* was tested (Fig 2A). Whereas cells transfected with control dsRNA facilitated robust Hh-induced reporter gene activation, cells transfected with *smo 5’UTR* dsRNA did not. Expression of a wild type *smo* cDNA lacking UTR sequence into the knockdown background fully rescued Hh-mediated reporter induction in this assay. dSmoNQ5 failed to rescue the Hh response, suggesting that loss of glycosylation renders dSmo incapable of signaling to induce Hh target gene expression.

In response to Hh stimulation, dSmo translocates from intracellular endosomes to the plasma membrane where it signals to its downstream effectors [9,11]. To determine whether dSmoNQ5 could traffic to an active location, Myc epitope-tagged WT or NQ5 dSmo proteins were co-expressed with the Calreticulin-EGFP-KDEL ER marker (GFP-ER) in Cl8 cells in the absence and presence of Hh (Fig 2B and [10,38]). In the absence of Hh, wild type dSmo localized to intracellular puncta that did not overlap with the GFP-ER marker and transitioned to the plasma membrane in the presence of Hh (Fig 2B, upper panels). dSmoNQ5 demonstrated a different localization pattern, overlapping almost completely with the ER marker in the absence and presence of Hh (Fig 2B, lower panels). Co-localization of GFP-ER with V5 epitope tagged ER resident proteins dBiP, dCnx and dCrc confirmed specificity of the ER marker protein (Fig 2B’). ER retention suggests that dSmoNQ5 might experience protein folding defects, an outcome that is consistent with the established role of glycosylation in protein folding in the ER [37].

Hh signaling plays an essential role in patterning vein structure and size of the adult *Drosophila* wing [39]. To assess whether glycosylation-deficient dSmo would function *in vivo*, the ability of dSmoNQ5 to rescue the phenotype induced by expression of dsRNA targeting the 3’ UTR of the endogenous *smo* gene was tested (*smo* ^*3’UTR*^, [40]). Dicer was co-expressed with the UTR dsRNA transgene to enhance dsRNA processing to yield a stronger phenotype [40]. When expressed with *UAS-dicer* under control of the *nubbin-GAL4* driver, *UAS-smo* ^*3’UTR*^ triggered disruption of vein patterning in the central region of the wing, indicative of attenuated Hh signaling (Fig 2C and 2E). Co-expression of a transgene encoding EGFP did not trigger a wing phenotype on its own and could not rescue the *smo* knockdown vein phenotype, thereby confirming that introduction of a third transgene into the *nub>Dcr; smo* ^*3’UTRdsRNA*^ background does not impact GAL4 activity (Fig 2F). Conversely, expression of wild type dSmo fully rescued the phenotype, and triggered modest ectopic vein formation anterior to longitudinal vein 3 (Fig 2G). Like EGFP, Co-expression of dSmoNQ5 in the dsRNA background failed to rescue the patterning defect (Fig 2H). To confirm that failure to rescue was not the result of lower expression of the NQ5 protein, lysates were prepared from wing imaginal discs and Smo protein was analyzed by western blot. dSmo levels were similar between wild type and NQ5-expressing flies (Fig 2I, compare lanes 2–3). Thus, N-linked glycans are required for dSmo function *in vivo*.

**Fig 3:**
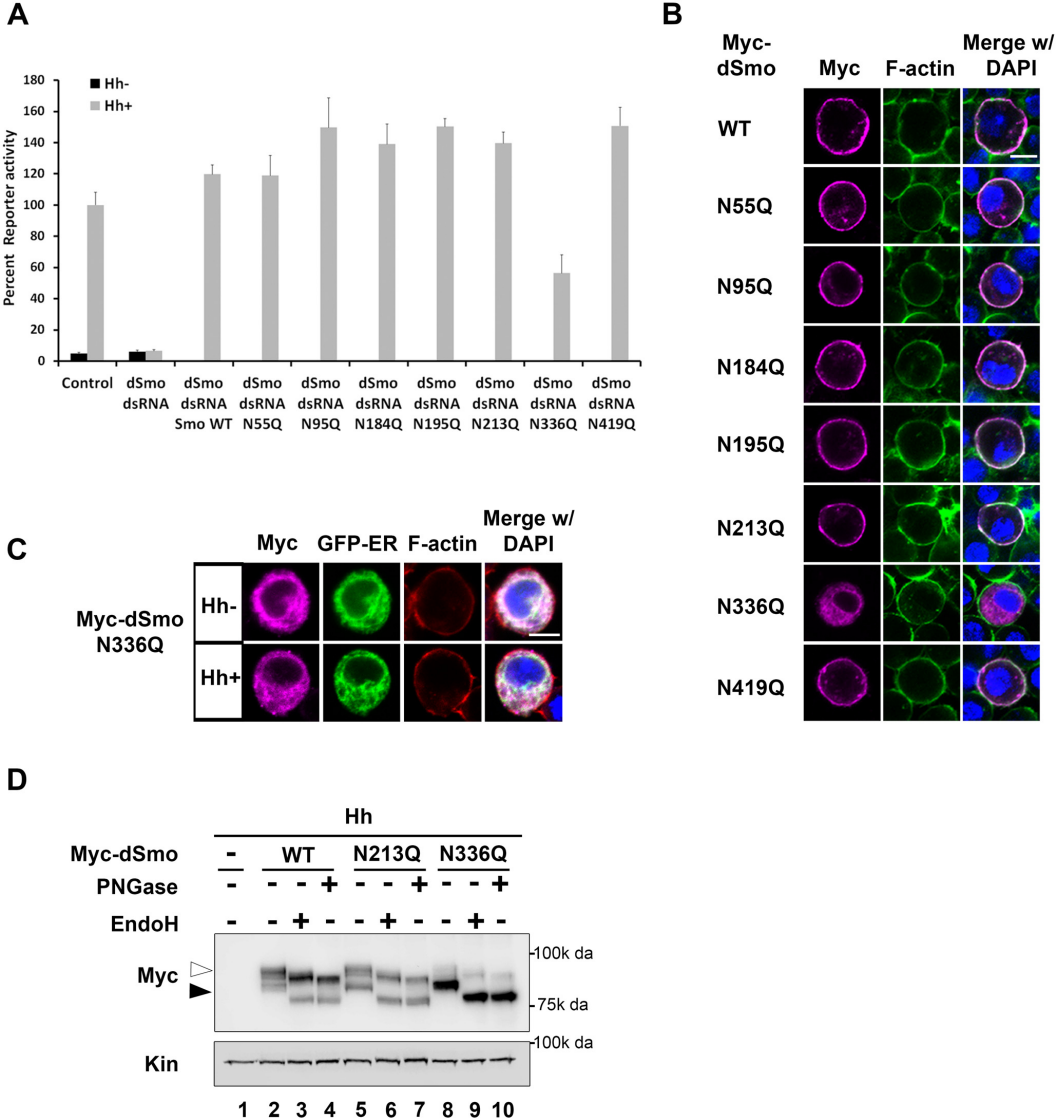
N336Q impacts dSmo trafficking and activity. **A.** N336 possesses an essential glycan modification. The rescue reporter assay was performed in *smo* knockdown Cl8 cells as in Fig 2. Each of the single N to Q mutants was able to rescue reporter gene activity in the *smo* knockdown background with the exception of N336Q. This mutant rescued to ~50% of the wild type activity. **B.** dSmoN336Q is mislocalized. Cl8 cells expressing Hh and the indicated dSmo proteins were imaged by immunofluorescence microscopy. Whereas each of the active single glycosylation site N to Q mutants (anti-Myc, magenta) reached the plasma membrane (indicated by F-actin stain, green) in Hh-expressing cells, dSmoN336Q failed to do so. DAPI (blue) marks the nucleus. Scale bar is 5 μm (upper right). **C.** SmoN336Q is retained in the ER. Myc-SmoN336Q expressed in Cl8 cells overlaps with the ER marker protein Calreticulin-KDEL-GFP. Scale bar is 5 μm (upper right). **D.** Treatment with deglycosylating enzymes demonstrates that the majority of dSmoN336Q is present in the EndoH sensitive, ER resident fraction (black arrowhead). WT and N213Q proteins are equally distributed between ER and post-ER fractions (white arrowhead).

To identify the essential sites of N-linked glycosylation contributing to dSmo ER exit and signaling, rescue reporter assays and sub-cellular localization analyses were performed for the single site N to Q mutants (Fig 3). Each of the single glycosylation site mutants was able to rescue reporter gene induction in the *smo* knockdown background similarly to wild type, with the exception of dSmoN336Q. This mutant demonstrated a ~50% reduction in activity, despite it expressing at higher protein levels (Figs 1D and 3A). Furthermore, dSmoN336Q was the only single glycan mutant that failed to accumulate on the plasma membrane in response to Hh, and instead, accumulated predominantly in the ER (Fig 3B and 3C). Enrichment of N336Q in the ER is also supported by western blot; wild type and N213Q, which reach the plasma membrane, were equally distributed between ER (black arrowhead) and post-ER fractions (white arrowhead). N336Q enriched in the EndoH sensitive ER-resident fraction (Fig 3D, lane 8, black arrowhead).

**Fig 4:**
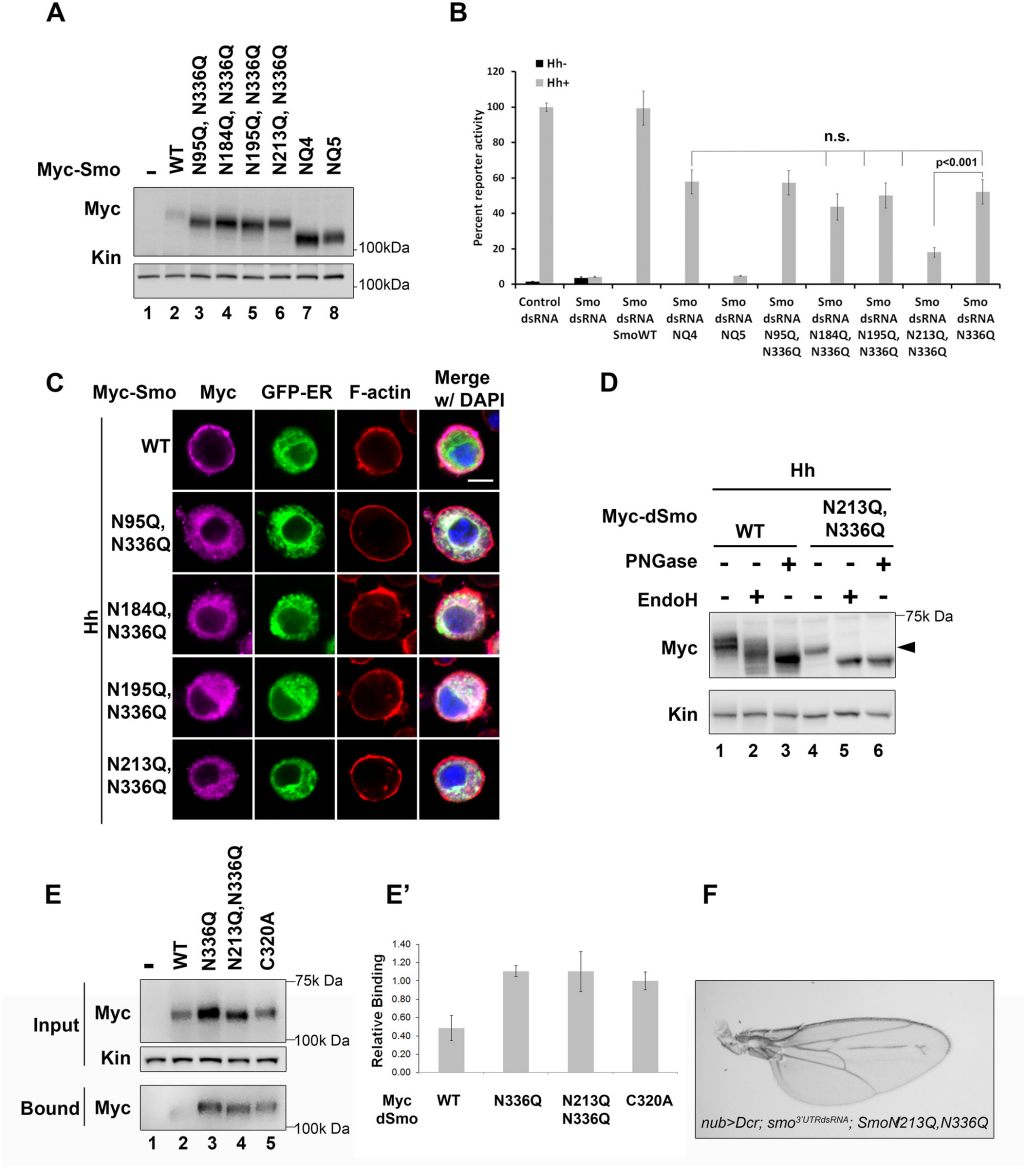
N213 glycosylation partially compensates for N336 N-glycan loss. **A.** The indicated mutants were expressed in Cl8 cells. Each of the mutant proteins migrated more quickly in SDS-PAGE than wild type Smo, but not as quickly as SmoNQ4 or NQ5. **B.** Glycosylation at N213 partially compensates for N336 glycan loss. The rescue reporter assay was performed as described in Fig 2A. Each of the indicated double mutants, with the exception of N213Q,N336Q, was able to rescue reporter gene expression in the *smo* knockdown background to a level similar to that of N336Q. dSmoN213Q,N336Q demonstrated a level of activity similar to dSmoNQ5. Significance was determined using Student’s t-test. **C.** N336Q-containing double mutants are retained in the ER. Cl8 cells expressing the indicated Smo proteins (anti-Myc, magenta), the Cal-EGFP-KDEL marker (green) and Hh were examined by immunofluorescence microscopy. Whereas wild type Smo reached the plasma membrane, the double mutants overlapped with the ER marker. ActinRed (red) marks F-actin. DAPI (blue) marks the nucleus. Scale bar is 5 μm (upper right). **D.** Treatment of lysates from WT or N213Q,N336Q expressing cells with deglycosylating enzymes reveals that the N213,336Q mutant is present in the EndoH sensitive ER fraction, arrowhead. **E-E’**. N336Q and N213Q,N336Q mutants have disulfide bond defects. Biotin-maleimide was used to tag free thiol groups in cellular lysates prepared from Cl8 cells expressing WT, N336Q, N213Q,N336Q and C320A dSmo proteins. WT dSmo is not well captured on NeutrAvadin beads (lane 2, bound). N-glycan mutants are captured similarly to the disulfide bond mutant C320A (lanes 3–5, bound), indicating that at least one disulfide bridge is disrupted by N-glycan loss. E’ shows the ratio of bound to unbound dSmo proteins normalized to kinesin. Relative binding was determined by densitometry analysis of two independent binding assays. C320A, which has an established disulfide bond defect served as positive control. It’s binding ratio was arbitrarily set to 1.0 and other values are shown relative to it. Error bars are provided to show the standard deviation between the two experiments. **F.** dSmoN213Q,N336Q fails to rescue *smo* knockdown *in vivo*. *UAS-dsmoN213Q*, *N336Q* was co-expressed with *UAS-dicer* and *UAS-smo* ^*3’UTR*^ using the *nubbin-Gal4* driver. Its expression did not modify the *smo* knockdown phenotype (compare to Fig 2F). Multiple progeny were analyzed over two crosses and a representative wing is shown.

The dSmoN336Q signaling defect was less pronounced than the signal loss observed for dSmoNQ5 (Fig 2A compared to Fig 3A), suggesting that a glycan modification on another residue may partially compensate for N336 glycan loss. To determine whether we could exacerbate the signaling defect observed for N336Q and identify the compensatory glycosylated residue, we generated double mutants harboring N336Q in combination with N to Q alterations for each of the additional conserved sites (N95, N184, N195 and N213, Fig 4A). Although altered migration of single N to Q mutations was modest (Fig 1D), each of the double mutants migrated more quickly through SDS-PAGE gels than wild type dSmo, but not as rapidly as dSmoNQ4 (N95Q, N184Q, N195Q, N213Q) or dSmoNQ5 (Fig 4A). Functional assays revealed that N95Q,N336Q, N184Q,N336Q and N195Q,N336Q induced an attenuated signal to downstream effectors that was similar to the dSmoN336Q response (Fig 4B). Conversely, dSmoN213Q,N336Q failed to effectively rescue reporter gene induction, demonstrating a significantly reduced activity level that was closer to that of dSmoNQ5 (Fig 4B). Like dSmoN336Q, each of the N336Q-containing double mutants failed to efficiently reach the cell surface in response to Hh, and instead enriched in the EndoH-sensitive ER fraction (Fig 4C and 4D, arrowhead).

N-linked glycans can impact disulfide bond formation during protein folding by recruiting ER chaperones and disulfide bond machinery [37,41]. It is therefore possible that ER retention of N-glycan dSmo mutants results from protein folding defects triggered by disulfide bond loss. To test whether disulfide bond formation was affected by N336Q or N213Q,N336Q mutation, lysates were prepared from cells expressing wild type or mutant dSmo proteins, and unpaired cysteines were detected by tagging free thiol groups with biotin-maleimide. Tagged proteins were captured on NeutrAvidin agarose and bound proteins were assessed by western blot and densitometry analysis [10]. Whereas dSmoWT was not effectively captured on NeutrAvadin agarose, both N336Q and N336Q,N213Q dSmo proteins were (Fig 4E, input vs. bound lanes 2–4). Averaged densitometry of two independent binding assays showed that N-glycan mutants were captured on the beads similarly to a dSmo protein harboring a C320A mutation, which disrupts the conserved disulfide bridge between the CRD and EC1 [10,42] (Fig 4E’). This suggests at least one disulfide bond is disrupted by loss of the sugar modification on N336, which is consistent with the glycan mutant failing to fold properly. Accordingly, *UAS-dSmoN213Q*, *N336Q* was unable to rescue the *smo* ^*3’UTR*^-induced phenotype *in vivo* (Fig 4F compared to Fig 2F). This failure was not the result of diminished protein levels, as evidenced by the abundance of dSmoN213Q,N336Q in imaginal disc lysates (Fig 2I, lane 3). Taken together, these results suggest that N336 is likely the site of an essential glycan modification that contributes to dSmo folding and ER exit, and that the N-linked glycan on N213 can partially compensate for its loss.

### Glycan modification is not required for mSmo folding or trafficking

**Fig 5:**
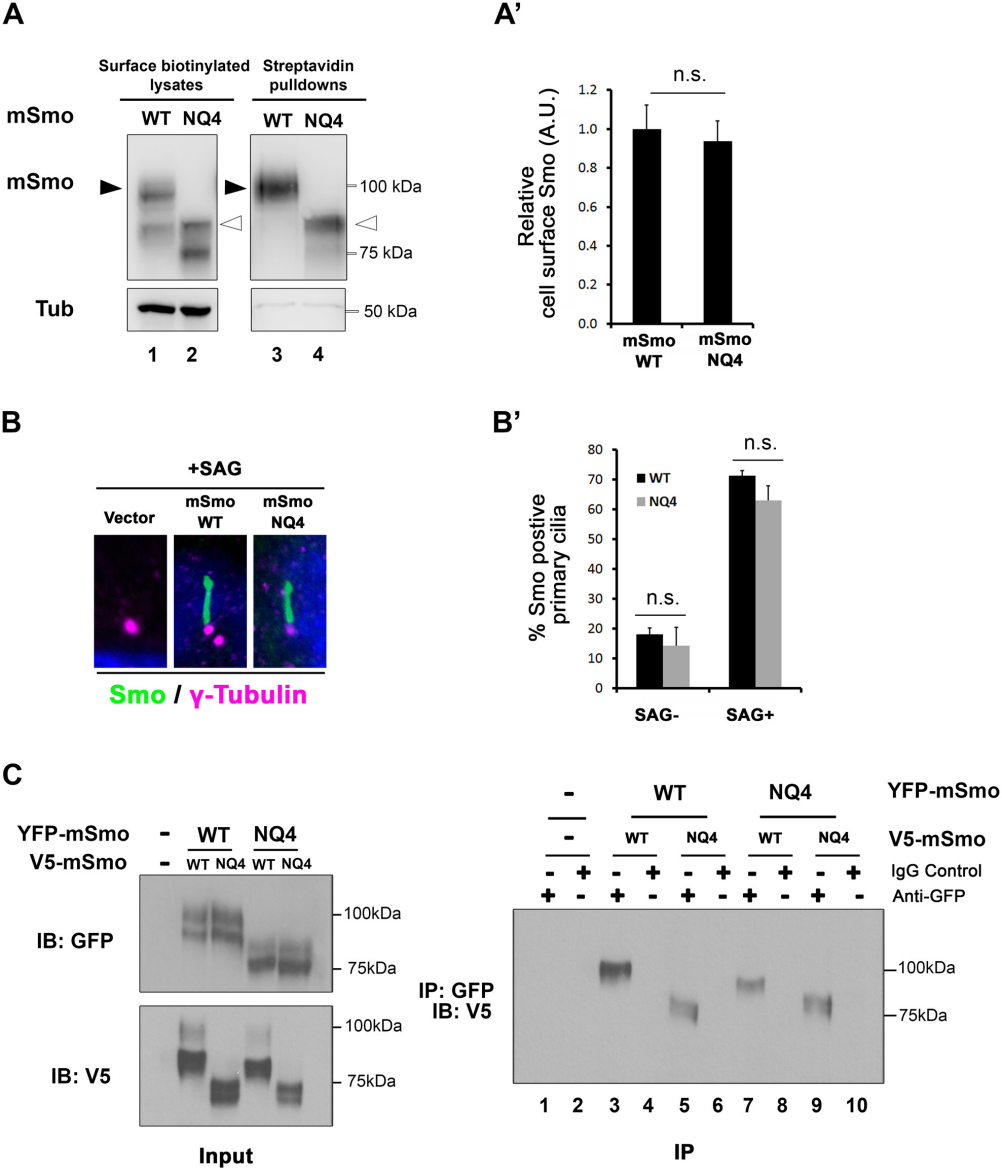
mSmo trafficking and dimerization are unaffected by N-glycan loss. **A.** mSmoNQ4 reaches the cell surface. Cell surface biotinylation analysis was performed on *Smo-/-* cells stably expressing WT or NQ4 mSmo proteins. Biotinylated proteins were collected on streptavidin beads, and analyzed by western blot and densitometry. Arrowheads indicate post-ER species that were used for quantification (black, WT and white, mutant). The ratio of extracellular to intracellular post-ER mSmo is similar for WT and NQ4 proteins (quantified in A’). The experiment was performed 5 times and all data pooled. Error bars indicate s.e.m. **B.** mSmoNQ4 localizes to the primary cilium. *Smo-/-* cells stably expressing WT or NQ4 Smo proteins (green, anti-Smo) were treated with SAG. Both WT and mutant mSmo proteins enrich in the primary cilium in response to SAG. γ-tubulin marks the basal body (magenta, anti-γ-tubulin) and DAPI marks the nucleus (blue). B’ shows quantification of ciliary localization (~100 cells over 3 independent experiments). **C.** NQ4 forms homo- and hetero-dimers. Differentially tagged WT and NQ4 mSmo proteins were expressed in NIH3T3 cells as indicated (left panel). mSmo complexes were immunoprecipitated (IP) from cellular lysates using anti-GFP antibody and immunocomplexes were analyzed by SDS-PAGE and western blot against the V5 epitope tag (right panel). mSmoNQ4-V5 co-purified with both WT and NQ4 YFP-tagged proteins.

To determine whether the essential role of N-glycans in Smo ER exit was conserved in vertebrates, *Smo-/-* cells stably expressing wild type or NQ4 mutant protein were used to examine Smo cell surface and ciliary localization (Fig 5A and 5B). To assess mSmoNQ4 plasma membrane localization, cell surface biotinylation was performed, and the ratio of surface-labeled to intracellular mSmo was determined by western blot and densitometry analysis. The ratios for WT and NQ4 mSmo were comparable, suggesting that N-glycosylation is not essential for mSmo trafficking to the plasma membrane (Fig 5A and quantified in A’). Upon agonist stimulation, mSmo enters the primary cilium as a requisite step in vertebrate pathway induction [7,43]. This trafficking capability was tested by treating mSmoWT or mSmoNQ4 stable cells with the Smo agonist SAG. Like the wild type protein, mSmoNQ4 enriched in the primary cilium in response to SAG treatment, indicating that the ligand-induced trafficking response is also intact (Fig 5B and quantified in B’).

Smo functions as an obligate dimer that assembles into higher-order oligomers upon Hh stimulation [44,45]. To determine whether the NQ4 mutant existed in a conformation that was permissive for dimer formation, wild type and NQ4 mSmo proteins harboring YFP or V5 epitope tags were generated. Differentially tagged mSmo proteins were co-expressed in NIH3T3 cells, immunopurified from cell lysates using GFP antibody and immunoblotted using anti-V5 (Fig 5C). mSmoNQ4-V5 immunoprecipitated with mSmoWT-YFP (right panel, lanes 5 and 7) and mSmoNQ4-YFP (right panel, lane 9), indicating that loss of N-linked glycosylation does not impact mSmo dimerization. Taken together, these results suggest that, contrary to what was observed for the *Drosophila* protein, loss of glycosylation does not negatively impact mSmo folding or trafficking.

### mSmoNQ4 binds ligands and induces canonical Sonic Hedgehog signaling

**Fig 6:**
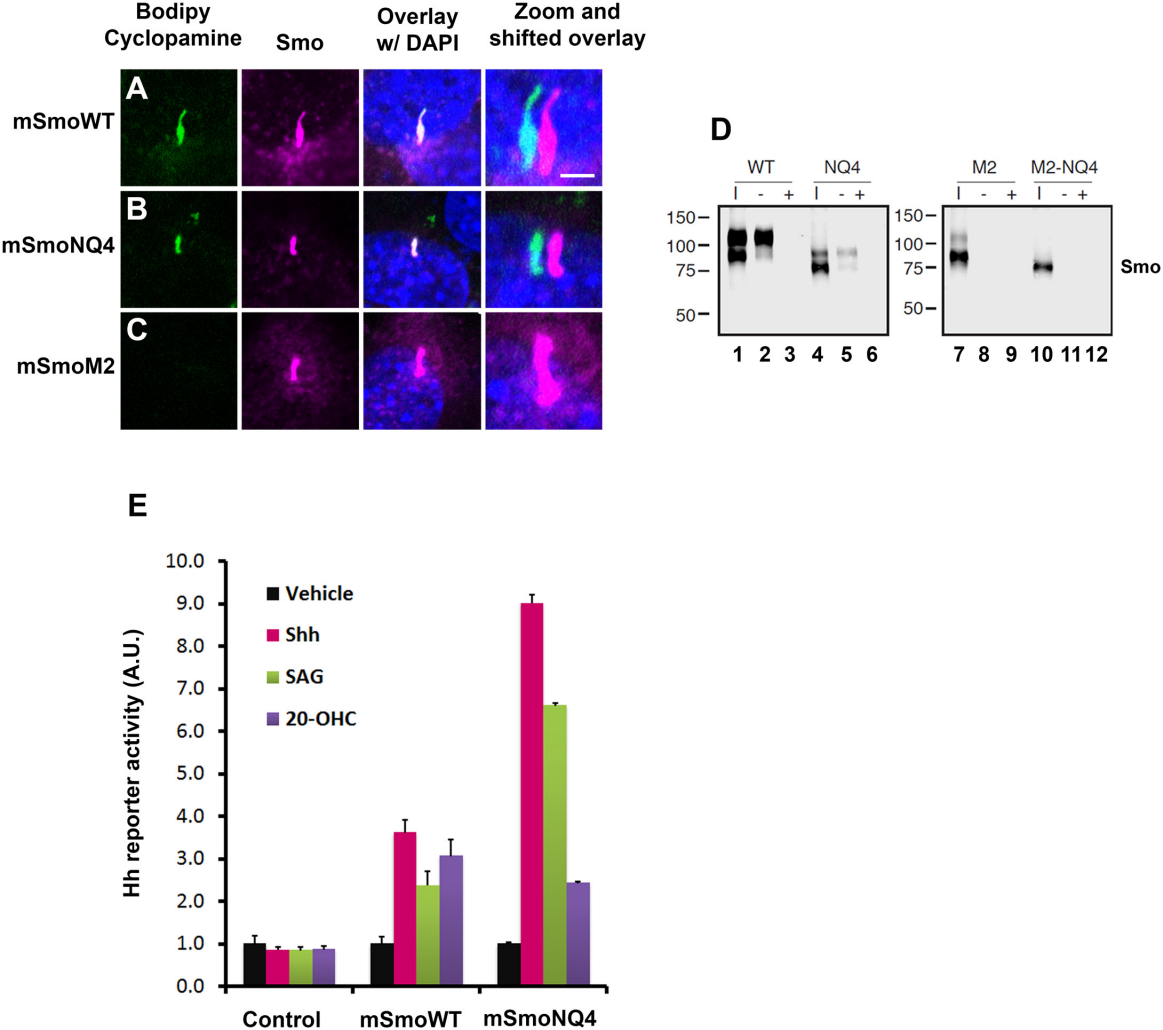
mSmoNQ4 binds ligands and activates canonical signaling. **A-C.** NQ4 binds bodipy cyclopamine. NIH3T3 cells expressing WT, NQ4 or M2 Smo proteins were treated with bodipy cyclopamine (5nM) for 4 hours, then fixed and immuno-stained for Smo (anti-Smo, magenta). Bodipy cyclopamine (green) accumulated in primary cilia in cells expressing WT or NQ4 Smo proteins. Bodipy cyclopamine failed to enrich in primary cilia in cells expressing oncogenic SmoM2. Higher resolution images of primary cilia with shifted channel overlays are shown (right). DAPI (blue) marks the nucleus. Scale bar is 5 μm (upper right). **D.** SmoNQ4 binds 20(S)-OHC. Lysates from cells expressing WT, NQ4, M2 or M2-NQ4 Smo proteins were incubated with 20(S)-OHC sepharose beads, and proteins purifying on the beads were analyzed by western blot using anti-Smo. Both WT (lane 2) and NQ4 (lane 5) Smo proteins were captured on 20(S)-OHC beads. Binding was specific, as it could be disrupted by 50 μM cold competitor (+). Neither SmoM2 (lane 8) nor SmoM2-NQ4 (lane 11) were captured on 20(S)-OHC beads. I indicates input. **E.** mSmoNQ4 signals to Gli. *Smo-/-* cells stably transfected with vector control (*pMSCV-puro*), *pMSCV-mSmoWT* or *pMSCV-mSmoNQ4* were transfected with the *8xglibs-luciferase* reporter and *tk-renilla* control. Both wild type and NQ4 Smo proteins induced reporter gene activity in response to Shh conditioned media (pink), SAG (green, 100 nM) and 20(S)-OHC (purple, 10 μM). Reporter assays were performed 2 times in triplicate and all data pooled. Error bars indicate s.e.m.

Smo has been reported to possess at least two distinct ligand binding pockets [15,46,47]. These include the 7-TM bundle, which binds SAG and cyclopamine, and an allosteric site in the CRD, which binds 20(S)-OHC [46,48,49]. Based upon the ability of SAG and cyclopamine to compete with each other for binding to Smo, their binding pockets are predicted to overlap [14,50]. As such, we used fluorescently labeled bodipy cyclopamine as a SAG surrogate to test functionality of the 7-TM binding pocket of mSmoNQ4 (Fig 6A and 6C). Like wild type mSmo, mSmoNQ4 bound and concentrated bodipy cyclopamine in the primary cilium (Fig 6A and 6B). Oncogenic SmoM2 signals independently of ligand and is refractory to cyclopamine inhibition [14]. Accordingly, mSmoM2 did not enrich bodipy cyclopamine in the primary cilium (Fig 6C). This result confirms the specificity of ciliary enrichment of the compound through binding to wild type and NQ4 mSmo proteins.

To determine whether mSmoNQ4 could bind the allosteric regulator 20(S)-OHC through its CRD, we expressed wild type or NQ4 mSmo proteins in 293T cells, and tested their ability to be captured on 20(S)-OHC conjugated sepharose beads (Fig 6D). Like the wild type protein, mSmoNQ4 was captured on 20(S)-OHC beads, indicating that the CRD ligand binding pocket is competent to associate with this small-molecule modulator (Fig 6D, lanes 2 and 5). Binding was largely restricted to the post-ER pool of mSmo, as the fully glycosylated form of wild type mSmo and the O-glycosylated form of NQ4 were preferentially enriched on 20(S)-OHC beads (Fig 6D, lanes 2 and 5 upper bands). Binding specificity was confirmed by competition with 50 μM 20(S)-OHC, which fully competed both mSmo proteins from the sterol beads (lanes 3 and 6). Similar to what we observed with bodipy cyclopamine, neither mSmoM2 nor mSmoM2NQ4 could be captured on the sterol beads (Fig 6D, lanes 8 and 11). Taken together with the above results, these binding studies suggest that N-glycosylation is dispensable for mSmo folding, trafficking, dimerization and ligand binding.

Smo has been reported to signal through G protein-dependent and -independent mechanisms to induce a range of cellular responses [23,51,52]. Although a subject of some debate, Smo is thought to signal primarily through G protein-independent mechanisms to induce target gene expression by Gli transcriptional effectors [23]. To determine whether signaling to Gli was compromised by loss of N-glycosylation, the ability of mSmoNQ4 to induce reporter gene activation in response to 20(S)-OHC, SAG and Shh conditioned media was tested. The *8Xglibs-luciferase* [53] reporter gene and *TK-renilla* control were transfected into mSmoWT, mSmoNQ4 and control stable lines, and reporter gene induction in response to Shh conditioned media, SAG and 20(S)-OHC was measured (Fig 6E). Both wild type and NQ4 mSmo cells induced reporter gene expression following exposure to Shh conditioned media, SAG or 20(S)-OHC (Fig 6E). Intriguingly, while both mSmo stable lines showed a similar Gli response following 20-(S)-OHC treatment, NQ4 appeared significantly more responsive to Shh and SAG than the wild type protein. Although we do not know the reason for the differential agonist responses by NQ4, we do not think poor binding is the cause of the blunted 20(S)-OHC response, as mSmoNQ4 can be captured on 20(S)-OHC beads (Fig 6D). Despite this, the robust Shh and SAG-induced responses suggest that NQ4 is highly sensitive for induction of canonical signaling to Gli.

### Non-canonical mSmo signaling is attenuated by N-glycan loss

**Fig 7:**
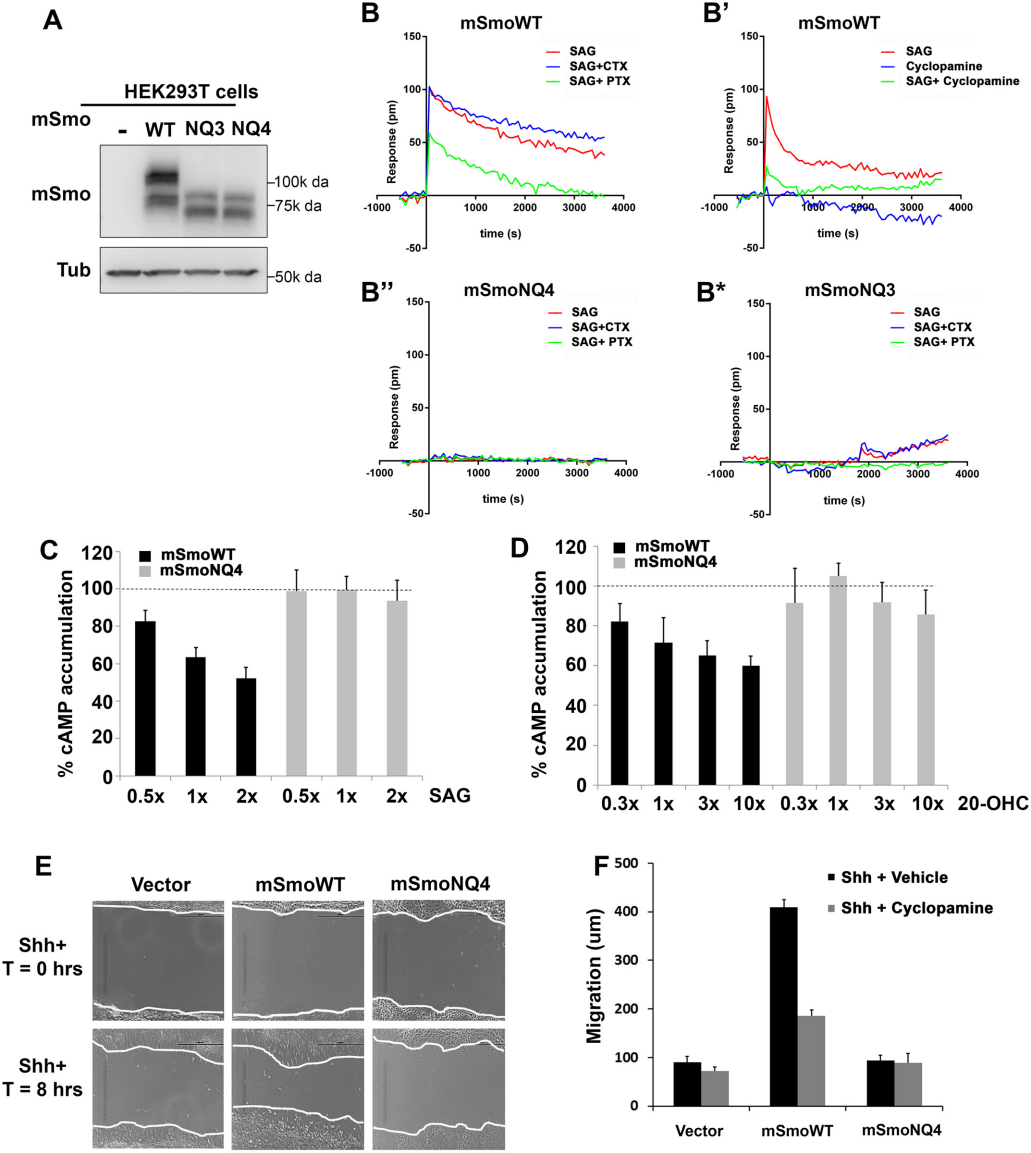
Glycosylation is required for non-canonical mSmo signaling. **A-B.** Glycosylation alters mSmo DMR. HEK293T cells expressing wild type (B-B’), NQ4 (B”) or NQ3 (B*) mSmo proteins were subjected to label-free DMR analysis. SAG- and cyclopamine-induced changes in cellular responses are displayed as refractive index alterations (Δ picometer). DMR responses were collected for cells pretreated with vehicle (red line, all panels), cyclopamine (B’, green line, 2μM), cholera toxin (CTX, blue line, B, B”, B*, 10 ng/mL) or pertussis toxin (PTX, green line, B, B”, B*, 10 ng/mL) prior to addition of 200 nM SAG. DMR experiments were performed 6–12 times. Representative graphs are shown. **C-D**. cAMP modulation is attenuated by NQ4 mutation. HEK293T cells expressing WT (black bars) or NQ4 (gray bars) mSmo proteins were pretreated with 0.5 μM forskolin prior to addition of SAG or 20(S)-OHC. cAMP levels were measured using HTRF with the LANCE Ultra cAMP kit. The forskolin induced cAMP level was set to 100% (dashed lines). 1x = 100 nM of the indicated ligand. Experiments were performed 6–8 times and all data pooled. Error bars represent s.e.m. **E-F.** Shh-regulated cell migration is attenuated by NQ4 mutation. Scratch assays were performed using *Smo-/-* cells stably transfected with *pMSCV-puro*, *pMSCV-mSmoWT* or *pMSCV-mSmoNQ4*. Migration into the scratch zone is shown at T = 0 and T = 8 hours. Shh conditioned media was added at T = 0. The non-canonical signal-regulated migration response was attenuated by N-glycan loss (quantified in F). SmoWT-induced migration was attenuated by cyclopamine treatment (F).

To assess whether NQ4 could induce the non-canonical signal through Gαi, we performed label-free dynamic mass redistribution (DMR) experiments using mSmoWT- or mSmoNQ4-expressing HEK293T cells. This cell line has previously been demonstrated to be competent for the Smo-Gαi signaling axis, and in our hands expresses WT and mutant mSmo proteins similarly (Fig 7A and [54–56]). DMR employs a biosensor to track real time ligand-induced changes in cell biomass resulting from cell shape alteration and/or redistribution of intracellular material. Optimized protocols for monitoring GPCR activity by DMR have been established, and can be used to examine direct agonist and antagonist-induced responses through all classes of heterotrimeric G proteins (Please see [54,57] for a comprehensive description). Because SAG induced a more robust reporter gene response by mSmoNQ4, we chose to use SAG in DMR assays. Consistent with its capacity to activate Gαi [58], mSmoWT induced a robust positive DMR response that was blunted by pretreating cells with Gαi-inactivating pertussis toxin (PTX, Fig 7B, red vs. green). The SAG-induced mSmoWT DMR was not affected by pretreating with Gαs-targeting cholera toxin (CTX, Fig 7B, blue), confirming that the response is specific to Gαi heterotrimeric G proteins. The positive SAG-induced DMR could also be attenuated by treatment with 2 μM cyclopamine (Fig 7B’, green), which is consistent with higher concentrations of cyclopamine being needed to compete with SAG for Smo binding [59]. Cyclopamine has been reported to function as a partial agonist for non-canonical Smo signaling in adipocytes to influence Warburg-like metabolism [60]. Cyclopamine effects on mSmoWT were therefore examined in the absence of SAG (Fig 7B’). Cyclopamine triggered a modest negative DMR response, indicating a change in basal mSmo signaling distinct from that induced by SAG (Fig 7B’ blue and S2A Fig). The shift was reversed by PTX treatment, suggesting that it is Gαi dependent, and may represent the PTX-sensitive partial agonism that has previously been reported (S2A Fig, blue vs. red) [60].

Examination of mSmoNQ4 Gαi signaling activity revealed a significant difference in the capacity of wild type and glycan-deficient proteins in mounting a non-canonical response. NQ4 failed to induce a measurable DMR in response to SAG or cyclopamine, suggesting a compromised Gαi signaling arm (Fig 7B” and S2B Fig). To confirm that attenuation of the DMR signature was not due to altered G protein engagement by mutation of the IC3 asparagine, we performed DMR using mSmoNQ3 in which the IC3 asparagine is not altered. Like mSmoNQ4, mSmoNQ3 failed to induce a robust DMR in response to SAG or cyclopamine, which is consistent with loss of extracellular glycosylation compromising non-canonical signal output (Fig 7B* and S2C Fig). To further test this, ligand-induced cellular cAMP modulation was measured in mSmo-expressing HEK293T cells. Cells were pretreated with forskolin to raise basal cAMP, and the ability of SAG or 20(S)-OHC to decrease cAMP was monitored (Fig 7C and 7D). Cells expressing mSmoWT triggered a dose-dependent reduction in intracellular cAMP following SAG or 20(S)-OHC stimulation (Fig 7C and 7D, black bars). Conversely, cAMP levels were not efficiently decreased in cells expressing mSmoNQ4 in response to these agonists (gray bars) further supporting that SmoNQ4 is compromised in its ability to signal through Gαi.

A documented non-canonical cell biological Smo response is its ability to influence cell migration via activation of RhoA and Rac GTPases [24]. To determine whether this non-canonical activity would be impacted by glycan loss, *Smo-/-* cells stably expressing mSmoWT, mSmoNQ4 or stably transfected with vector control were subjected to a scratch test, and their ability to migrate through the scratch following Shh ligand treatment was quantified (Fig 7E and 7F). Consistent with Smo being required for Shh-induced cell migration, vector transfected *Smo-/-* cells demonstrated a modest 8 hour migration that was not significantly affected by cyclopamine treatment (Fig 7E and 7F). Conversely, Shh-treated *Smo-/-* cells stably expressing WT mSmo showed an obvious migration into the scratch zone, traveling on average 4-fold further than vector control cells (Fig 7E and 7F). This migration was attenuated by cyclopamine treatment, indicating that the Gαi-mediated response is sensitive to cyclopamine inhibition, as was suggested by our DMR analysis (Fig 7B’ and 7F). Conversely, mSmoNQ4-expressing cells failed to efficiently migrate into the scratch zone over the 8 hour assay window (Fig 7E and 7F). The inability of SmoNQ4 to mount a strong non-canonical Gαi response, as evidenced by attenuated DMR, cAMP reduction and migration capacity, are in direct contrast to the robust SAG- and Shh-mediated Gli responses elicited by mSmoNQ4 (Fig 6E). Taken together, these results suggest an intrinsic bias of the under-glycosylated Smo protein toward the canonical signaling arm.

## Discussion

In this study we examined the predicted N-linked glycosylation motifs in *Drosophila* and mouse Smo proteins, and identified residues harboring glycan modifications contributing to function. Consistent with the differing number and localizations of N-glycan acceptor sites between the two Smo proteins, and the noted differences between fly and vertebrate glycosylation processes [35,36], a clear difference in the role of N-glycan modification for fly and mouse Smo signaling was revealed.

### Glycosylation in *Drosophila* Smo signaling

Drosophila Smo Signaling was impacted by loss of N-linked glycosylation at N336 in EC1, and was further compromised by introducing a second mutation at N213 in the CRD. The combined loss of these two N-linked glycosylation acceptor sites recapitulated the loss of activity observed for dSmoNQ5, which harbors mutations of the five sites that are conserved across sequenced *Drosophila* Smo proteins. Disruption of glycosylation at these two sites triggered disulfide bond loss and ER retention, suggesting an essential role for these post-translational modifications during dSmo protein folding.

Glycosylation plays a range of roles in membrane and secretory protein biology including trafficking, stability, structural rigidity, functionality and most commonly, protein folding in the ER [37]. Glycan modifications are frequently localized to regions where protein secondary structure changes and stabilizing influences are needed [37,61]. Accordingly, N-linked glycans can impact the formation of stabilizing disulfide bonds through binding to the ER chaperones Calnexin and Calreticulin. Glycan-bound chaperones then recruit ERp57 to the client protein to drive disulfide bond formation [37,41]. Consistent with this essential functionality, N336 of Smo is adjacent to a conserved disulfide bond that is formed between TM3 residue C339 and EC2 residue C413 [10,42]. The functional effects of disrupting this disulfide bridge in flies ranges from robust ligand-independent signaling activity when the C339 bond partner is targeted, to weak hypomorphic activity when the C413 partner is affected [10,62]. SmoN336Q signaling activity is reduced by ~50%, which is consistent with the loss of signal observed with a C413A mutation [10,62]. As such, we speculate that glycosylation at N336 serves as a linchpin for dSmo protein folding by contributing to formation of this essential disulfide bridge. Accordingly, N336Q mutation resulted in an increase in free thiols, which is consistent with disruption of at least one disulfide bond.

A theme that is common to glycobiology that was recapitulated with dSmo is the observation that a single glycan modification may not be necessary when targeted individually, but when disrupted along with a second modification, becomes essential [37]. We observed this for N213, a glycan acceptor site localized to the amino-terminal CRD. When mutated alone, this residue failed to impact dSmo function, but when targeted along with N336, ablated Smo signaling. Like N336, N213 is localized adjacent to a cysteine engaged in a disulfide bond that is required for Smo functionality [42,47]. This disulfide bond occurs between C218 and C238 of the *D*. *melanogaster* Smo protein and assists in positioning the amino-terminal CRD toward the EC loop domains [42]. This conformation has been postulated to affect the binding of small-molecule Smo modulators, as its disruption severely compromises Smo signaling [47].

Despite some under-glycosylated dSmo mutants being retained in the ER, their protein levels appeared significantly higher than wild type, suggesting that the mutants are not misfolded enough to be actively targeted for ER associated degradation (ERAD). We have observed similar behavior by other dSmo mutants affecting the TM3-EC2 disulfide bond; C339A and C413A dSmo mutants also accumulate in the ER and escape ERAD under basal conditions [10,36]. A potential explanation for the accumulation of ER-retained dSmo mutants is that their failure to efficiently exit the ER removes them from the normal recycling/degradation circuit [11,63,64]. In the absence of Hh, dSmo that reaches the plasma membrane is subject to ubiquitination and Shibiri-mediated endocytosis, resulting in its eventual degradation [63,64]. Mutant protein that lingers in the ER is removed from this regulatory cycle, thereby allowing for its accumulation. Consistent with this hypothesis, we found that ER-retained SmoNQ5 demonstrated a significantly longer half-life than the wild type protein. Combined, these results support an essential role for two N-linked glycosylation sites in dSmo, N213 and N336, thereby broadening the knowledge about post-translational modifications contributing to *Drosophila* Smo functionality.

### Glycosylation in murine Smo signaling

**Fig 8:**
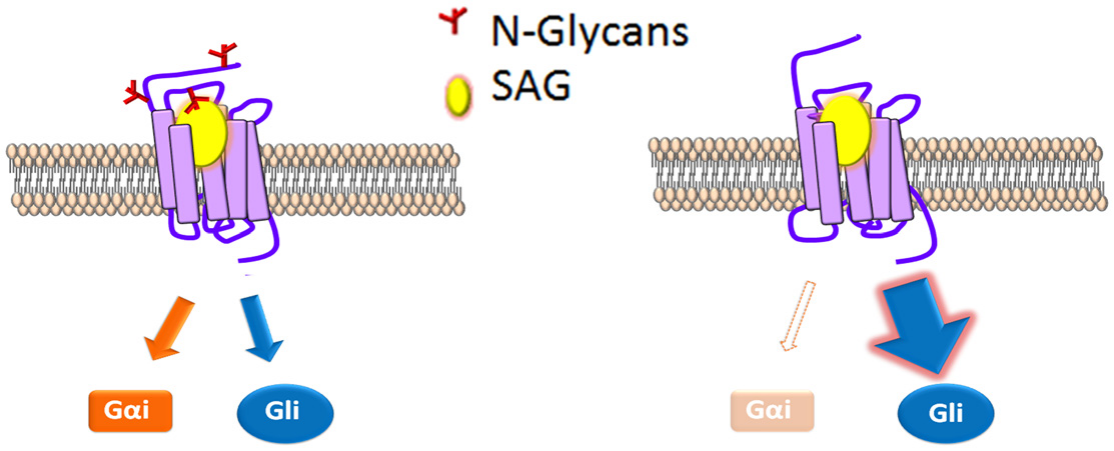
N-glycosylation status correlates with signal bias. A model for modulation of mSmo signal bias. SAG (yellow) binding to N-glycosylated mSmo stabilizes a conformation that effectively signals through both canonical and non-canonical routes. SAG binding to the mSmo mutant stripped of N-glycans (red) silences non-canonical signaling, but is highly permissive for canonical signaling.

Contrary to the essential role we uncovered for N-linked glycans in dSmo ER exit and signaling, the mSmo glycosylation mutant demonstrated normal trafficking, dimerization, ligand binding and canonical signaling to Gli transcriptional effectors. However, a clear difference was evident in the ability of mSmoNQ4 to induce the non-canonical signal via Gαi. Although biased agonism was initially thought to result only from synthetic ligands, it is now believed that signal bias can be facilitated through natural ligands, and may serve as a mechanism to diversify GPCR signaling capability [26,28,29]. Consistent with this model, a recent study demonstrated that distinct chemokines targeting the same chemokine receptor could elicit clear differences in G protein-dependent and -independent signals [29]. The exact mechanism(s) by which individual ligands induce differential signal output from the same receptor are not yet clear. However, ligand binding to allosteric sites on a given receptor likely stabilizes different active receptor conformations that may result in varying signal efficiencies to distinct routes [28,29,65]. We therefore hypothesize that the Gli signal bias induced by stripping mSmo of its N-linked glycosylation results from a change in its agonist-stabilized conformation. Rather than being in a conformation that is equally competent for both signals, the glycan-stripped protein is stabilized in a conformation more permissive for the canonical signal route (Fig 8). Consistent with this, mSmoNQ4 showed significantly enhanced SAG- and Shh-induced Gli responses compared to those of the wild type protein. Intriguingly, both WT and NQ4 showed a similar 20(S)-OHC Gli response. One explanation for this could be that the NQ4 conformation shifts induced by SAG and 20(S)-OHC binding differ, with the SAG shift being more permissive for Gli activation. However, SAG and Shh induced a similar enhanced NQ4 Gli response, leading us to favor an alternate interpretation. The endogenous Smo ligand controlled by Ptch has not yet been established, but is suggested to be an oxysterol-like molecule [15,46,49]. It is therefore possible that availability of 20(S)-OHC to mSmo may be influenced by Ptch, which would account for the similar 20(S)-OHC responses observed.

The predicted N-linked glycosylation sites are localized near to the known mSmo ligand binding pockets; N1 and N2 flank the CRD, which binds 20(S)-OHC and N4 localizes to EC3, which is believed to assist in coordinating small molecule binding to the 7-TM core [42,46,48,49,66]. As such, context-specific changes in modification of these sites could alter ligand-induced responses, and may be one mechanism by which signal axis specification occurs in nature. Although we did not observe changes in the Smo glycosylation pattern in response to pathway activation in our assays, it is possible that chronic Hh stimulation might lead to changes in glycosylation that impact signal output over time. To our knowledge, there are only two examples of N-linked glycans influencing signal bias; 1) glycosylation of Protease-activated receptor 1 governs bias between G_q_ vs. G_12/13_ signal output [67], and 2) differential glycosylation of follicle stimulating hormone (FSH) dictates signal bias of the FSH receptor [68,69]. We therefore propose that signal bias is a new functionality to be added to the list of GPCR processes affected by N-linked glycosylation, and that vertebrate Smo may have evolved to exploit this functionality as it acquired non-canonical signaling capabilities.

## Materials and Methods

### Sequence analysis

The Clustal W2 program was used to align human (UniProt Q99835), mouse (UniProt P56726), rat (UniProt P97698), chicken (UniProt O42224), zebrafish (UniProt Q90X26) and the following *Drosophila* Smo protein sequences: *D*.*melanogaster* (FBgn0003444), *D*. *simulans* (FBgn0194405), *D*. *sechellia* (FBgn0171642), *D*. *erecta* (FBgn0116840) *D*. *yakuba* (FBgn0234200), *D*. *ananassae* (FBgn0097636), *D*. *persimilis* (FBgn0163503), *D*. *pseudoobscura* (FBgn0243541), *D*. *mojavensis* (FBgn0139780), *D*. *willistoni* (FBgn0217303), *D*. *virilis* (FBgn0211743) and *D*. *grimshawi* (FBgn0119026). Glycosylation sites were predicted using NetNGlc prediction software at www.cbs.dtu.dk/services/NetNGlyc/. The Smo snake plots were generated with the transmembrane topology prediction tool Protter (http://wlab.ethz.ch/protter/start).

### DNA constructs and antibodies

*pCDNA-V5-mSmo* was generated by introducing DNA sequence encoding the V5 epitope tag (GKPIPNPLLGLDST) after amino acid 51 in *pCDNA-mSmo [36]* using Phusion site directed mutagenesis kit (Thermo Scientific). Asn (N) to Gln (Q) mutagenesis was performed on Drosophila and mouse Smo expression vectors using QuikChange II Mutagenesis Kit (Stratagene). dBip, dCnx and dCrc expression plasmids were generated by cloning the respective cDNAs obtained from the DGRC into *pAc5*.*1* (Life technologies). *pCS2-YFP-mSmo*, the *pAc-Cal-KDEL* ER marker and *pAc-Myc-dSmo* were described previously [10,38,70].

Antibodies used for western blot analyses include anti-mSmo (SCBT), anti-GFP (Cell Signaling), anti-V5 (Life technologies), anti-kinesin (Cytoskeleton, Inc.), anti-alpha tubulin (cell signaling) and anti-Myc (Sigma) and anti-SmoC [15].

### Cell culture, transfection and cellular lysate preparation

All cells were grown at 37°C in an atmosphere of 5% CO_2_ in DMEM supplemented with 10% heat inactivated FCS or FBS (HEK293T), 0.1 mM nonessential amino acids, 2 mM L-glutamine, 1% Pen-Strep and 1mM sodium pyruvate. For *Smo-/-* cells FCS was not heat inactivated.

To generate stable lines from *Smo-/-* cells, ecotropic retroviruses were produced by cotransfection of retroviral expression plasmids (*pMSCV-puro*, *pMSCV-puro-mSmo* or *pMSCV-puro-mSmo* ^*NQ4*^) with packaging plasmids (*pMD-old-Gag-Pol* and *pCAG4-Eco*) into HEK293T cells using FuGene 6 (Roche Applied Bioscience). HEK293T cells were maintained in DMEM (Invitrogen, Grand Island, NY) supplemented with 10% FBS, Pen/Strep, 1mM Sodium Pyruvate and 1mM L-Glutamine. Approximately 3x10^6^ cells were plated the day before transfection on 10cm dishes. The next day cells were transfected with 12μg vector DNA, 6μg *pMD-old-gag-pol* and 2μg *CAG4-Eco* using 60μl of Fugene6. Twenty-four hours post-transfection media was removed and replaced. Six hours later viral supernatant was harvested, replaced and incubated overnight. In the morning viral supernatant was harvested and the procedure was repeated. Viral supernatants were filtered using a 0.45 micron filter prior to use. For viral transduction 1 x 10^6^ *Smo-/-* cells were seeded ~24 hours prior to viral transduction. Cells were then incubated in media containing 20ug/ml polybrene (American Bioanalytical, Inc., Natick, MA) plus viral supernatants. The selection process was started ~48 hours post incubation using Puromycin (Invitrogen, Grand Island, NY).

For biochemical assays in NIH3T3 cells, the indicated cell types were seeded at a density of 1 x10^6^ cells/60 mm dish, then transfected the following day with 3 μg of the indicated mSmo expression vectors using Lipofectamine 2000 or 3000 (Invitrogen) or Fugene6 (Promega). Cell lysates were prepared ~48 hours post transfection in RIPA buffer (50 mM Tris-HCl (pH 7.4), 150 mM NaCl, 0.25% deoxycholic acid, 1% NP-40, 1 mM EDTA, 0.1% SDS, 0.5 mM DTT, and 1× PIC (Roche)) as described [36].

For Drosophila biochemical analyses, ~3X10^6^ Cl8 cells were plated and transfected the following day with 3 μg of *pAc-hh* and 5μg of the indicated dSmo expression vectors using Lipofectamine 2000 (Invitrogen). DNA content was normalized with *pAc5*.*1A* empty vector. Cellular lysates were prepared ~48 hours post-transfection using NP-40 lysis buffer (1% NP-40, 150 mM NaCl, 50 mM Tris, 50 mM NaF, 0.5 mM DTT, and 1X PIC (Roche), pH 8.0) and centrifuged at 2000 x g for 10 minutes. Resulting supernatants were used in assays.

### Deglycosylation and phosphatase treatments

Cellular lysates were treated with 1000U peptide-N-glycosidase F (PNGase), 1000U endoglycosidase H (EndoH) or 800U O-glycosidase + 100U Neuraminidase for two hours at room temperature prior to SDS-PAGE and western blot. All enzymes were obtained from New England Biolabs (NEB). Lambda phosphatase treatment was preformed exactly as previously described [36]. Experiments were performed a minimum of three times and representative blots are shown.

### Half-life assessment

Cl8 cells transfected with WT or NQ5 dSmo expression vectors were treated with 25 μg/ml cycloheximide (Calbiochem) and incubated for the indicated time prior to harvesting and lysis in NP-40 buffer. Protein samples were analyzed by western blot and Myc-Smo protein levels relative to Kin were determined by densitometry analysis using Photoshop CS4. Data were plotted as percent signal relative to the 0 time point to determine half-life. The experiment was performed three times and representative results are shown.

### Maleimide labeling

Maleimide labeling of free thiols was performed on lysates from Cl8 cells expressing the indicated dSmo protein as we have previously described [10]. Labeling experiments were repeated twice without Hh and once with Hh. Similar results were obtained and a representative western blot is shown. Densitometry analysis was performed on the two Hh(-) experiments using Photoshop CS4. The graph represents the pixel intensity ratio of bound to unbound protein normalized to kinesin. Error bars indicate standard deviation, and were provided to illustrate the minimal variation between the two experiments.

### Co-immunoprecipitation analysis

RIPA lysates were prepared from NIH3T3 cells expressing the indicated mSmo proteins. Approximately 200 μg of total protein was pre-cleared against 20 μL of equilibrated protein A/G plus agarose beads (50% slurry, SCBT). Cleared lysates were incubated with 5 μg anti-GFP or rabbit IgG control with rocking at 4°C for 2 hours. Immune complexes were collected on protein A/G beads for 45 minutes with gentle rocking at 4°C. Beads were washed twice in lysis buffer and associated proteins were extracted in 2x sample buffer (2% w/v SDS, 2 mM DTT, 4% v/v glycerol, 0.04 M Tris-HCL, pH 6.8 and 0.01% w/v Bromphenol blue) and analyzed by SDS-PAGE and western blot.

### Cell surface biotinylation

*Smo-/-* cells stably expressing mSmoWT or mSmoNQ4 were seeded at a density of ~2x10^6^ cells/100 mm dish and allowed to grow for 24 hours. Cells were then incubated overnight in culture media supplemented with 0.5% FCS plus 100 nM SAG. Forty-eight hours post seeding, live cells were washed three times with cold 1x phosphate buffered saline (PBS) pH 7.4 and then incubated with gentle shaking for 30 minutes at 4°C in 2 mL of 1x PBS containing 0.5 mg/ml EZ-Link Sulfo-NHS-Biotin (Pierce). Biotinylation was quenched by washing cells twice with cold 1x PBS containing 50 mM Tris. Cells were harvested and lysed in RIPA buffer. Lysates were incubated with 50 μl of Streptavidin agarose beads (Thermo Scientific) for 1 hour at 4°C. Beads were spun down and supernatants were collected. Beads were washed three times with RIPA buffer and bead purified proteins were extracted in sample buffer. Proteins from the supernatant and Streptavidin-purified beads were analyzed by SDS-PAGE and western blot. Densitometry analysis was performed using Photoshop CS4 and the ratio of signal densities for Streptavidin-bound cell surface vs. intracellular Smo was determined. The experiment was repeated three times and all data pooled. Error bars indicate s.e.m. The western blot is representative.

### Ligand binding

NIH3T3 cells were transiently transfected with the indicated mSmo expression vectors. Forty-eight hours post transfection, live cells were incubated for 4 hours at 37°C in serum-free media containing 5 nM bodipy cyclopamine (Toronto Research Chemicals Inc.). After three 10 minute washes in 1x PBS, cells were fixed and immuno-stained for mSmo as described [36]. Purification on 20(S)-OHC beads was performed as described [15,49].

### Reporter assays

For Drosophila reporter assays, ~3.75X10^5^ Cl8 cells were plated in 24-well dishes and transfected the following morning with 50 ng *ptcΔ136-luciferase*, 10 ng *pAc-renilla* normalization control, 50 ng *pAc-hh* and 50 ng *pAc-myc-smo*. DNA content was normalized with the empty vector *pAc5*.*1A*. Cell lysates were prepared in passive lysis buffer ~48 hours post-transfection, and luciferase activity was measured using Dual-Luciferase Reporter Assay System (Promega). All assays were performed two or three times in duplicate or triplicate, and all data pooled. Reporter activity is shown as the percent activity relative to the control Hh response, which was set to 100%. Error bars indicate standard error of mean (s.e.m.). Statistical significance was determined using the two tailed Student’s t-test.

For murine reporter assays approximately 300,000 *pMSCV-puro*, *pMSCV-mSmoWT* or *pMSCV-mSmoNQ4* stable cells were transfected with 200 ng *8Xglibs-luciferase* and 50 ng *pRL-TK* as previously described [15,49]. Approximately 16 hours post transfection, cells were incubated in serum free media for ~ 2 hours and then switched to 0.5% serum media containing SAG (100 nM), Shh conditioned media (300μl/ml) or 20(S)-OHC (30 μM) and incubated for another 24 hours before measuring reporter activity. Assays were performed two times in triplicate, and all data pooled. Error bars indicate s.e.m. Shh conditioned media was generated as described [14].

### DMR analysis

The DMR signature was determined using an EnSpire Multimode Plate Reader (PerkinElmer, Waltham, MA, USA) by label-free technology optimized for G-protein response as previously described [54,55]. Briefly, 24 hours before the assay, cells were seeded at a density of 7000 cells per well in 384-well sensor microplates. Cells were treated with vehicle, CTX or PTX (10 ng/mL) in cell culture medium overnight. Prior to taking readings, cells were washed twice with assay buffer (Hank’s Balanced Salt Solution (Life Technologies), 20 mM HEPES, 0.1% BSA, pH 7.5), then incubated for 2 hours at 24°C in assay buffer with 0.1% DMSO (vehicle) prior to scanning for a baseline optical signature. SAG was added to 200 nM and cyclopamine to 2 μM final concentration and DMR responses were monitored for 8000–15,000 seconds. Kinetic results were analyzed using EnSpire Workstation Software v 4.10.

### cAMP accumulation

Approximately 3500 HEK293T cells expressing the indicated mSmo proteins were plated per well in 384 well plates and pretreated for 10 minutes with 0.5 μM forskolin (Sigma) prior to adding SAG or 20(S)-OHC (1x = 100nM). cAMP levels were determined by using the LANCE Ultra cAMP kit (PerkinElmer) and a Pherastar FS Microplate Reader (BMG Labtech, Ortenberg, Germany).

### Immunofluorescence

*Drosophila* immunofluorescence analyses were performed exactly as previously described [36]. For detection of mSmo in the primary cilium, *Smo-/-* cells stably transfected with vector control, mSmoWT or mSmoNQ4 plasmids were incubated overnight in culture media supplemented with 0.5% FCS plus SAG (100 nM). The following morning cells were fixed in 4% paraformaldehyde for 15 minutes at room temperature followed by three 10 minute washes in 1xPBS. Fixed cells were permeabilized and blocked in PBGT (1xPBS+0.1% TritonX-100+5% Normal goat serum) for 1 hour, then incubated overnight with anti-Smo (SCBT) and anti- γ-tubulin (Sigma) antibodies. AlexaFluor 488 or 555 conjugated secondary antibodies (1:1000; Life technologies) were used. Slides were mounted using Vectashield with DAPI (Vector Labs). Immunofluorescence data were collected using Zeiss LSM 510 or 710 and images were processed using Zen2009 and Photoshop CS6. For all immunofluorescence experiments, multiple cells were examined over a minimum of three experiments, and representative images are shown. For quantification of ciliary localization, 96–126 cells were counted over three experiments and the percent of cells showing Smo in the primary cilium was determined. The paired t-test was used to determine statistical significance.

### Cell migration assays

Cell migration was assessed by performing scratch assays as previously described [24,71]. *Smo-/-* cells stably transfected with *mSmoWT*, *mSmoNQ4* or empty *pMSCV-puro* vector control were seeded in a 12 well dish at a density of 3x10^5^ cells/well and allowed to grow in complete media until fully confluent. Once cells reached confluency, they were incubated in media supplemented with 0.5% FCS overnight. The following morning (time T = 0), cells were washed with PBS and a scratch was made across the well using a p20 pipette tip. Scratched wells were washed twice in PBS to remove scratched cells, then incubated for 8 hours (T = 8) in low serum media supplemented with Shh conditioned media (1:4 ratio), 0.5 μM cyclopamine or vehicle control. For Shh + cyclopamine conditions, cyclopamine was added to the overnight incubation prior to scratching and adding Shh media. Images were taken at T = 0 and T = 8 using an EVOS cell imaging system (Life Technologies). In each experiment, three different zones along the scratch were imaged, and in each zone 10 equally spaced points were used for analysis. The experiment was performed three times and all data pooled for quantification. Distance migration was calculated and analyzed as previously described [24].

### Fly crosses

*UAS-myc-smo* transgenic flies were generated by targeting the respective transgenes to the landing site 3L-68E using the *PhiC31* system [72]. *UAS-smo* ^*3′UTR*^ *-dsRNA* and *UAS-Dicer*; *nubGAL4* fly lines were gift from David Hipfner [40]. Genotypes are indicated in figure labels. All crosses were performed at 29°C. Wings from adult females were mounted on glass slide using DPX mounting media and imaged using a Ziess LSM200-C microscope with a Ziess AxioCam ICc3 camera. Wings from multiple progeny over two independent crosses were analyzed and representative wings are shown. To test protein expression levels, ten wing imaginal discs for each genotype were isolated and lysed in 2x SDS sample buffer (4% SDS, 4 mM DTT, 8% glycerol, 0.08 M Tris-HCL, pH 6.8, 0.02% bromphenol blue). Lysates were analyzed by SDS-PAGE and western blot.

## Supporting Information

S1 Fig Identification of Smo N-glycosylation sites. **A-B.** The snake plots of the extracellular and transmembrane domains of *D*. *melanogaster* (A) and mouse (B) Smo proteins were generated using the Protter prediction tool. Predicted N-linked glycosylation sites are in blue and extracellular cysteines that are involved in disulfide bond formation are in green. The signal peptide is orange. **C.** Seven N-linked glycosylation sites were identified in *D*. *melanogaster* Smo, five of which are conserved across all sequenced *Drosophila* Smo proteins. Consensus sequences of the N-linked glycosylation motifs are highlighted in gray and Asn acceptor sites are in bold. Conserved cysteines that form disulfide bonds are indicated by arrowheads. Glycan acceptor sites for the *D*. *melanogaster* protein are numbered at top. **D**. dSmoNQ5 has an extended half-life. Western blots for dSmo (anti-Myc) and Kinesin (Kin) were performed on lysates from cycloheximide (CHX)-treated Cl8 cells expressing wild type or NQ5 Myc-Smo proteins (left panel). Densitometry analysis of Smo signal normalized against Kin revealed a clear increase in SmoNQ5 half-life (~4 hours NQ5 vs. ~1 hour WT, right panel). Data are plotted as percent remaining relative to the 0 time point. The experiment was repeated three times. A representative experiment is shown.
(TIF)

S2 Fig Glycosylation alters mSmo response to cyclopamine. **A**. HEK293T cells expressing wild type mSmo were subjected to label-free DMR analysis. SAG- and cyclopamine-induced changes in cellular responses are displayed as refractive index alterations (Δ picometer). DMR responses were collected for cells pretreated with vehicle (blue line) or pertussis toxin (PTX, red line) prior to addition of cyclopamine (2 μM). DMR experiments were performed 6 times. Representative graphs are shown. **B-C.** mSmoNQ4 and mSmoNQ3 proteins were treated with SAG (200nM, red), cyclopamine (2μM, blue) or SAG + cyclopamine (green). Both glycosylation deficient mutants failed to respond, showing similar DMRs under all three conditions.
(TIF)
