# Supplementary figures and images for: Functional Divergence in the Role of N-Linked Glycosylation in Smoothened Signaling
Source: PLoS Genet. 2015 Aug 20;11(8):e1005473. doi: 10.1371/journal.pgen.1005473 (PMC4546403; doi:10.1371/journal.pgen.1005473)

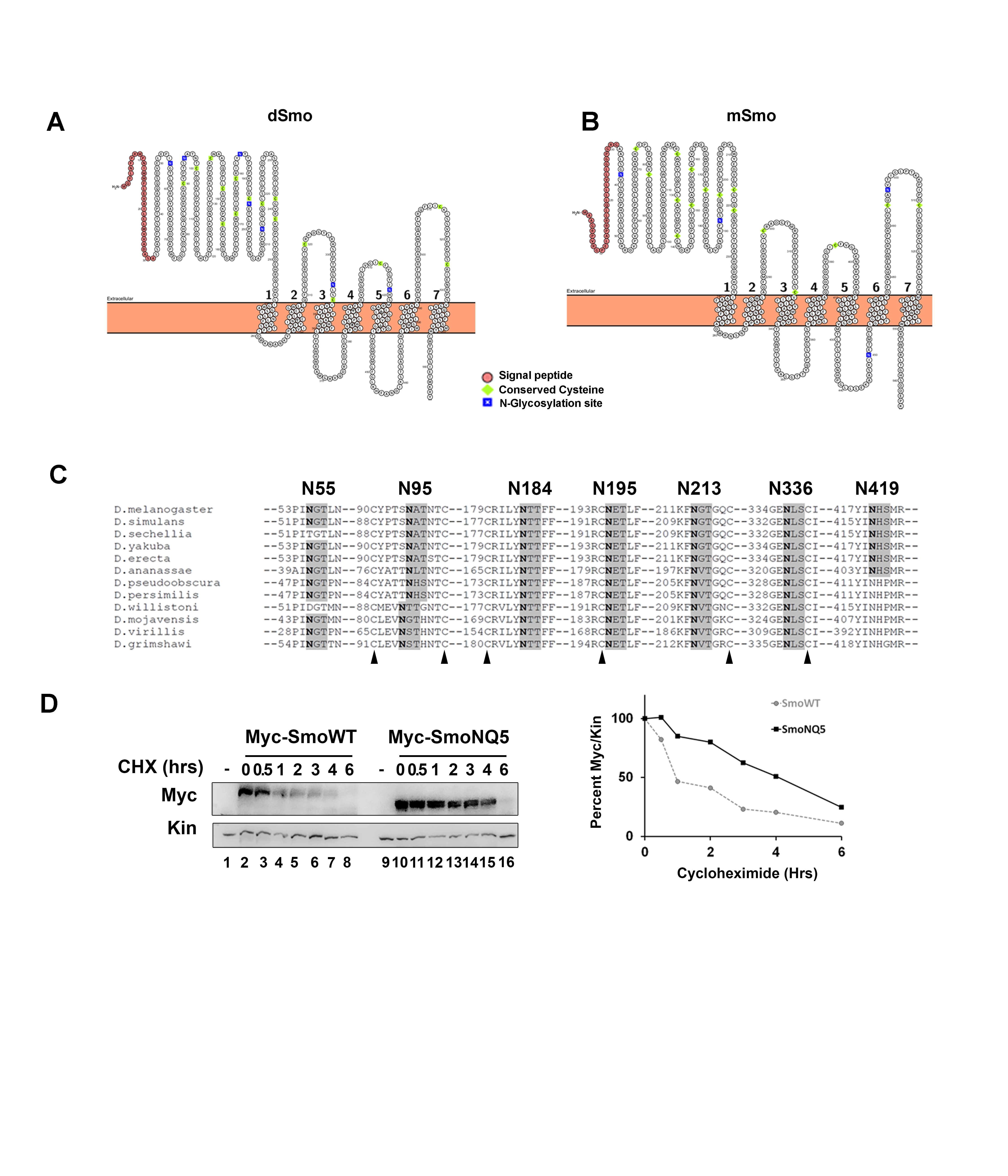

Supplement: S1 Fig — A-B. The snake plots of the extracellular and transmembrane domains of D. melanogaster (A) and mouse (B) Smo proteins were generated using the Protter prediction tool. Predicted N-linked glycosylation sites are in blue and extracellular cysteines that are involved in disulfide bond formation are in green. The signal peptide is orange. C. Seven N-linked glycosylation sites were identified in D. melanogaster Smo, five of which are conserved across all sequenced Drosophila Smo proteins. Consensus sequences of the N-linked glycosylation motifs are highlighted in gray and Asn acceptor sites are in bold. Conserved cysteines that form disulfide bonds are indicated by arrowheads. Glycan acceptor sites for the D. melanogaster protein are numbered at top. D. dSmoNQ5 has an extended half-life. Western blots for dSmo (anti-Myc) and Kinesin (Kin) were performed on lysates from cycloheximide (CHX)-treated Cl8 cells expressing wild type or NQ5 Myc-Smo proteins (left panel). Densitometry analysis of Smo signal normalized against Kin revealed a clear increase in SmoNQ5 half-life (~4 hours NQ5 vs. ~1 hour WT, right panel). Data are plotted as percent remaining relative to the 0 time point. The experiment was repeated three times. A representative experiment is shown. (TIF) [file pgen.1005473.s001.tif]

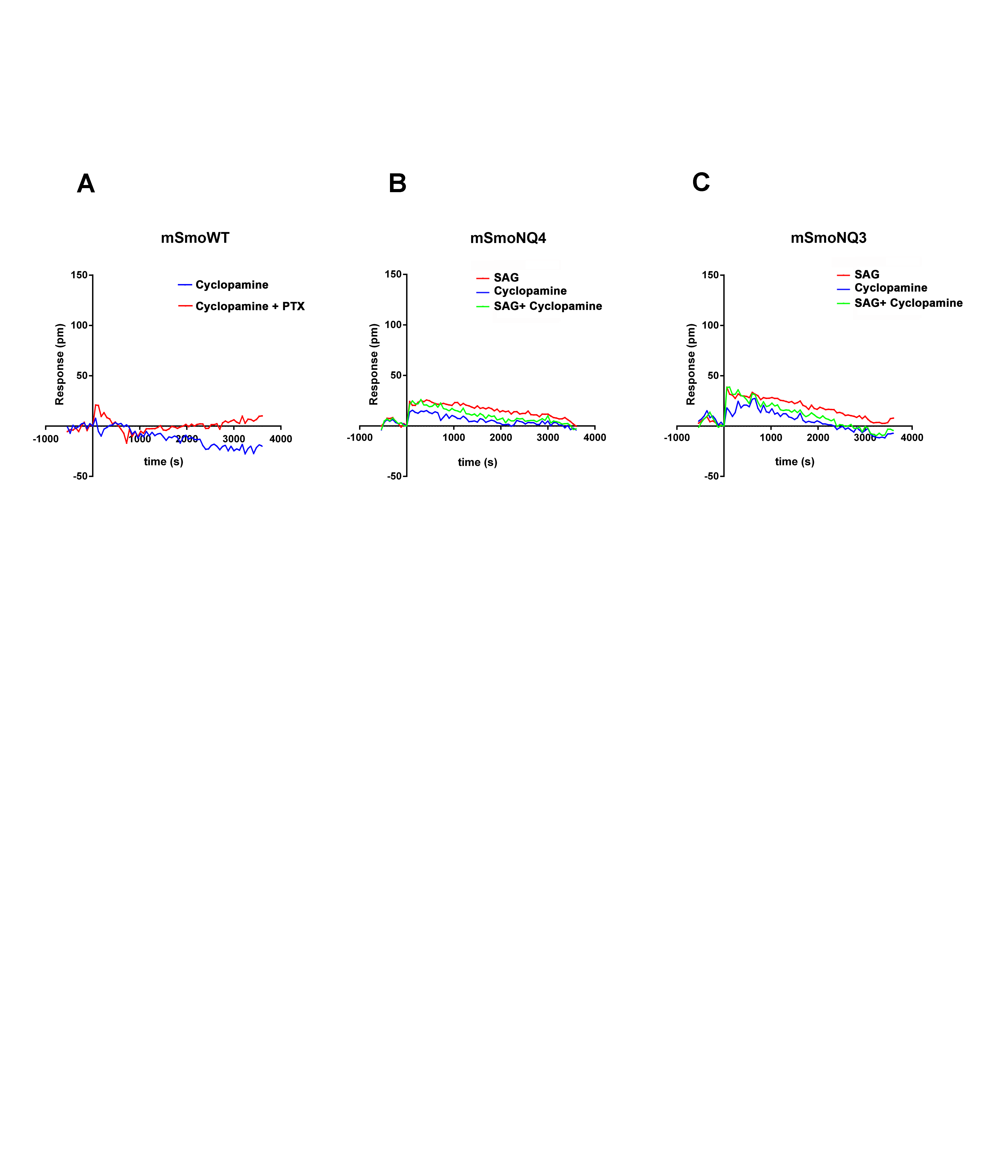

Supplement: S2 Fig — A. HEK293T cells expressing wild type mSmo were subjected to label-free DMR analysis. SAG- and cyclopamine-induced changes in cellular responses are displayed as refractive index alterations (Δ picometer). DMR responses were collected for cells pretreated with vehicle (blue line) or pertussis toxin (PTX, red line) prior to addition of cyclopamine (2 μM). DMR experiments were performed 6 times. Representative graphs are shown. B-C. mSmoNQ4 and mSmoNQ3 proteins were treated with SAG (200nM, red), cyclopamine (2μM, blue) or SAG + cyclopamine (green). Both glycosylation deficient mutants failed to respond, showing similar DMRs under all three conditions. (TIF) [file pgen.1005473.s002.tif]
